# Costs and benefits of early response in the Ebola virus disease outbreak in Sierra Leone

**DOI:** 10.1186/s12962-020-00207-x

**Published:** 2020-03-16

**Authors:** Klas Kellerborg, Werner Brouwer, Pieter van Baal

**Affiliations:** Erasmus School of Health Policy & Management, Erasmus University Rotterdam, Rotterdam, The Netherlands

**Keywords:** Cost-effectiveness, Ebola virus disease, Policy evaluation

## Abstract

**Background:**

The 2014–2016 Ebola virus disease (EVD) outbreak in West Africa was the largest EVD outbreak recorded, which has triggered calls for investments that would facilitate an even earlier response. This study aims to estimate the costs and health effects of earlier interventions in Sierra Leone.

**Methods:**

A deterministic and a stochastic compartment model describing the EVD outbreak was estimated using a variety of data sources. Costs and Disability-Adjusted Life Years were used to estimate and compare scenarios of earlier interventions.

**Results:**

Four weeks earlier interventions would have averted 10,257 (IQR 4353–18,813) cases and 8835 (IQR 3766–16,316) deaths. This implies 456 (IQR 194–841) thousand DALYs and 203 (IQR 87–374) million $US saved. The greatest losses occurred outside the healthcare sector.

**Conclusions:**

Earlier response in an Ebola outbreak saves lives and costs. Investments in healthcare system facilitating such responses are needed and can offer good value for money.

## Background

The West African Ebola virus disease (EVD) outbreak was the largest EVD outbreak since the virus was discovered. The outbreak mainly affected Guinea, Liberia, and Sierra Leone which together reported 28,616 confirmed, probable and suspected cases and 11,310 deaths [1]. Disruptive effects also affected health-seeking behavior and healthcare delivery [2–6]. As the case counts grew, the outbreak drew international attention. In August 2014 the WHO published the Roadmap for response, outlining three phases of response initiatives to combat the outbreak [7]. In October 2014, during the first phase, the UN Mission for Ebola Emergency Response (UNMEER) was launched [8]. UNMEER had several aims: that 70 percent of cases would be isolated and that 70 percent of the burials would be conducted in a safe manner. Approximately 2 months after the UNMEER initiated interventions were implemented, the national weekly case counts decreased [9]. Although the response operations seemed to effectively control the outbreak, critical voices raised an issue with the timeliness of the responses. Both the recognition of the outbreak and the implementation of the interventions came too late according to critics [10–12]. The EVD epidemic highlighted the importance of surveillance systems for early detection as the virus remained undetected for the first 3 months of the EVD outbreak [12–15].

Previous studies have estimated the effectiveness of various interventions, both real and hypothetical aimed at mitigating the outbreak [16–27]. In an early stage of the outbreak Rivers et al. explored several different interventions and found that those would not effectively control the outbreak [19]. Kucharski et al. estimated the number of averted cases due to the introduction of additional hospital beds in Sierra Leone, and found that the increased capacity averted approximately 56,000 cases [26]. Barbarossa et al. estimated the effect of the response efforts on the number of cases and concluded that a 5-week earlier implementation would halve the outbreak size [27]. Other studies have investigated the health effects of the EVD outbreak caused by disruption of the health care system [28–30]. Apart from interventions, the economic effect of the outbreak has also been studied [31–33]. Bartsch et al. performed a cost of illness study comprising EVD treatment costs and productivity losses, suggesting that the total cost of the epidemic in Sierra Leone was approximately 30 million US$ [31]. Additionally, Kirigia et al. estimated future production losses due to EVD mortality to approximately 60 million international$ in Sierra Leone [32]. Finally, The World Bank estimated the outbreaks’ impact on the GDP of the outbreak-affected economies affected to be 2.8 billion US$, where Sierra Leone was most affected and incurred a loss of 1.9 billion US$ [33].

Although studies have investigated the effects of the outbreak in different intervention scenarios little work has been performed on the combination of potential health benefits and cost savings of earlier interventions. In this paper, we focus on providing estimates of costs and health consequences of the outbreak and the potential benefits of an earlier response. Moreover, this study also provides relevant input for discussions on more general investments to strengthen relatively weak health systems [32]. To enable comparability, we measure health losses in Disability Adjusted Life Years (DALY) and take into account the costs associated with an outbreak both within and outside the healthcare sector. DALYs are a summary measure of health that comprise both length and quality of life [34], being widely used in cost-effectiveness studies which facilitates comparison with similar studies. Furthermore, DALYs lost because of early death are closely linked to productivity losses as health facilitates productivity. Given that the EVD outbreak affects people in their working age/productive years, an exclusive focus on the costs incurred within the health system would result in an incomplete picture of the impact of earlier response [35].

## Methods

To estimate the incremental health benefits and potential costs of earlier interventions in the scenario of the EVD outbreak in Sierra Leone we used a compartment model to describe the transmission under the baseline scenario- the actual outbreak -, and several counterfactual scenarios. The counterfactual scenarios mimic earlier interventions varying from 1 day earlier up to 4 weeks earlier. We attached treatment costs and production losses to the transmission model compartments. We also attached disability weights to the compartments, from which DALYs were calculated. The sum of costs and DALYs were calculated under the baseline and the two counterfactual scenarios. We assessed the uncertainty of our results with respect to the uncertainty surrounding input parameters and carried out a sensitivity analysis for several key parameters.

### Transmission model

**Fig. 1:**
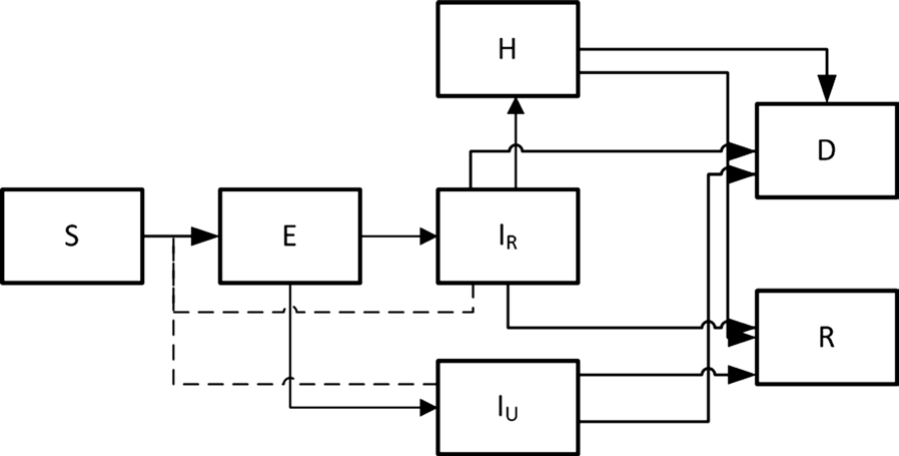
Compartment model schematic. Solid lines indicate transition paths; dashed lines indicate transmission routes. With the following compartments, Susceptible (S), Exposed (E), Infectious and reported (I_R_), Infectious and not reported (I_U_), Hospitalized (H), Dead (D) and lastly Recovered (R)

To explore the potential benefits of earlier response we used an extended SEIR compartment model, based on the model of Kucharski et al. [26]. The model aims at describing the natural course of the disease and incorporating setting specific context such as hospitalization in either holding centers or treatment centers, which is then run on a district level. Figure 1 depicts the model schematics: upon contracting the virus the individual leaves the Susceptible compartment (S) and enters the latent compartment (E). From the E compartment the individuals’ transition to the infectious compartment (I). When entering the I compartment, the individuals are infectious to others. As not all cases are assumed to be reported, the I compartment is differentiated in reported cases and cases not being reported. We assumed that the infection rate is the same for both I compartments and from there on infected individuals may die or recover from the EVD. If the infected individuals are reported then, if district beds are available, they are hospitalized. During hospitalization, they are assumed not to be infectious to others. During the outbreak, facilities with different functions existed such as holding centers and treatment centers. In our model we treated the different facilities as the same, assuming that the fatality rates did not differ. Within each district, homogenous mixing was assumed and no spatial interaction was accounted for. The whole population was assumed to be susceptible. Due to the small number of reported cases we excluded the Bonthe district.

The transmission rate and parameters capturing the effect of the interventions implemented during the outbreak were fitted to the reported number of cases by weighted least squares, from the WHO’s situation reports [1]. The parameters were fitted separately for each district, to reduce identifiability issues we derived some parameter values from other studies (see Additional files 1 and 2 for more information). In Table 1 the parameters used in the model that are not district dependent are presented.

**Table 1:** Parameters estimated and fixed with their respective source

Parameter	Description	Value	References
a_2_	Maximum value of transmission rate	Estimated	See Additional files 1 and 2
a_1_	Slope of transmission rate parameter	Estimated	
	Midpoint of transmission rate parameter	Estimated	
b_1_	Slope of intervention rate parameter	Estimated	
	Midpoint of intervention rate parameter	Estimated	
1/σ	Latent period	10.4 days	[36]
1/γ_CR_	Time to recovery in the community	11.7 days	[36]
1/γ_CD_	Time to death in the community	6.8 days	[36]
1/γ_HR_	Discharge rate	11.6 days	[36]
1/γ_HD_	Time to death for hospitalized	5.2 days	[36]
	Proportion reported	83%	[36]
1/*ω*	Time to notification	4.8 days	[36]
1/η	Hospitalization rate	4.6–1.3 days	See Additional files 1 and 2
δ_C_	Fatality rate in the community	91.9%	[36]
δ_H_	Fatality rate for hospitalized	60.3%	[36]

We allowed the infection rate to vary to accommodate different outbreak paces between districts. After the 1st of October 2014, the date of the UNMEER implementations [37], we introduced the effect of interventions in the model. We allowed the effect of the interventions to vary between districts. As the weekly number of reported cases declined at different speeds we did not force a linear decrease on the effect of the interventions.

### Translating morbidity and mortality effects into DALYs and costs

The health loss due to EVD expressed in DALYs is the sum of health losses during an illness and the health lost because of an early death. To estimate health losses we attached disability weights from the Global Burden of Disease (GBD) study to the relevant compartments [38]. Health losses because of early death were assumed to be equal to Health Adjusted Life Expectancy (HALE) estimates for Sierra Leone from GBD. To estimate the remaining HALE for each case the observed age distribution of reported cases was applied to the final outbreak size [23]. The full societal costs as a consequence of EVD include not only direct costs such as treatment costs for EVD but also indirect costs such as production losses, due to sickness and death at a young age. As in Bartsch et al. two treatment options were included: supportive and extensive supportive care [31]. Supportive care consists of paracetamol, oral rehydration salts, metoclopramide for nausea. Extensive care adds morphine for pain, diazepam for convulsions, Ringer’s lactate against shock and broad-spectrum antibiotics. As no proportion of the severity of cases was available a random number was drawn from a uniform distribution from 0 and 1 for each run representing the proportion of cases receiving supportive care. For treatment costs, the costs estimated by Bartsch et al. were used [31]. For reasons of international comparability, we calculated the production losses according to the Human capital method [39]. GDP per capita was used as a proxy for annual production losses and was multiplied by the HALE lost for early deaths to estimate lifetime production losses. An implicit assumption here is that life years spent in poor health do not result in productivity gains in our estimation. For recoveries, the productivity loss from Bartsch et al. due to absenteeism was used [31]. Costs are all expressed in 2014 US dollars (Table 2).

**Table 2:** Costs and health parameters included, mean and 95% confidence interval in brackets

Cost group	Age group:			References
	< 15 years	15–44 years	≥ 45 years	
Supportive care				
Patient recovers	431 (413–450)	446 (428–466)	447 (428–464)	[31]
Patient dies	178 (163–195)	185 (169–202)	185 (168–202)	[31]
Extensive supportive care				
Patient recovers	598 (576–622)	830 (800–862)	830 (801–859)	[31]
Patient dies	238 (217–259)	321 (292–351)	322 (291–351)	[31]
Personnel costs				
Patient recovers	59 (57–61)	59 (57–61)	59 (57–61)	[31]
Patient dies	21 (19–23)	21 (19–23)	21 (19–23)	[31]
Productivity losses due to				
Absenteeism during illness episode	23 (22–24)	23 (22–24)	23 (22–24)	[31]
Mortality	42 747.2 (12 355.9–128 273.4)	29 640 (7 599.2–90 040.3)	13 227.5 (2 934.1–42 393.5)	Calculated using the wealth distribution [40]
Disability weights				
Acute phase of illness	0.133 (0.088–0.19)			[38]
Post-sequelae	0.219 (0.148–0.308)			[38]
Mortality, HALE (range)	51.3 (48.11–53.51)	34 (24.76–43.84)	13.92 (7.32–21.38)	[38]
Duration of illness				
Acute phase, recover	15.1 (14.6–15.6) days			[38]
Acute phase, death	8.2 (7.9–8.4)			[38]
Post-sequelae	0.75 years (0.417–1.135)			[38]

By age groups and costs groups. Expressed in 2014 $US

### Interventions and counterfactual scenarios

To explore the potential benefits and costs of timely interventions we created counterfactual scenarios of earlier interventions. In our initial analysis we compare the baseline scenario—interventions as they were implemented by the UNMEER—to a counterfactual scenario of interventions taking place 4 weeks earlier. We then continued to investigate the effect on health and costs with interventions taking place between the baseline scenario and 4 weeks earlier in steps of 1 day. The counterfactual scenarios were modeled by moving the time of interventions in the transmission model 4 weeks earlier. This affected the transmission parameter and also the hospitalization rate and the case fatality rates for those hospitalized.

### Assessment of uncertainties of transmission models

We assessed the uncertainty of our outcomes by taking into account the uncertainty around the input parameters of the compartment model and our health and cost estimates. In our main scenario of a 4 week earlier counterfactual we implemented a stochastic model using the tau-leaping approximation of the Gillespie’s algorithm with a time step of 0.01 days [41, 42]. The approximation treats individuals as discrete units and translates the rates into probabilities allowing for stochasticity in all transitions. We performed several univariate sensitivity analysis to explore the impact of key input parameters on our outcomes. We varied the proportion of underreporting by ten percentage points, the time for cases to be reported, the time to hospitalization and the timing of interventions by 1 day each.

## Results

### Model fit

**Fig. 2:**
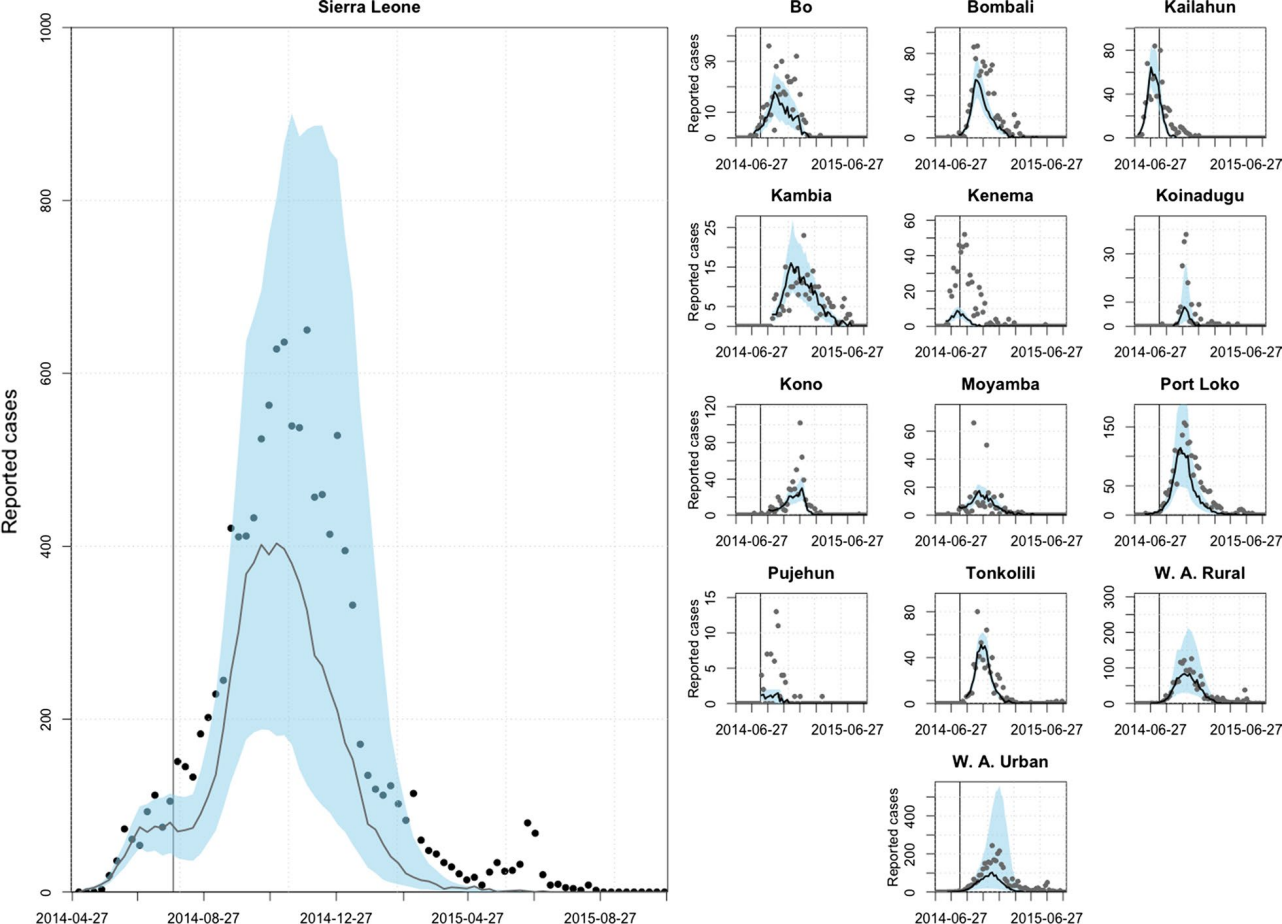
Stochastic model fit on the national and district level. Solid line shows the median number of reported cases of 1500 model runs. Blue areas are the interquartile range. Reported cases by the WHO patient database are given as black dots. Vertical line shows the date of implementation of interventions

Figure 2 shows the fit of the reported cases of the models median and interquartile range by district and nationally against the reported number of weekly cases. Our model estimated 8609 (3882–8609) reported cases which is a bit lower than the number actually of reported cases, with the largest discrepancy being in the Western Area Rural district reported cases. Distinct temporal differences between districts can be observed such as in Kailahun and Kenema, which experienced a peak of reported cases earlier than other districts. These two districts displayed a decrease in cases before the implementation of the UNMEER interventions. For the fitted parameter values per district and results per district, see Additional files 1 and 2.

### Effect of earlier interventions

Districts with a large number of cases and exponential growth showed the greatest savings of costs and health. In a large number of the districts, the time of interventions and the decrease of cases correlated well. Four weeks earlier interventions resulted in cost savings and health gains compared to the baseline scenario. The savings in both costs and health were largely due to the averted mortality as seen in Table 3 where results are shown based on outcomes of the stochastic model. Our result suggests that interventions implemented 4 weeks earlier would have halved both the costs and the health losses. Results by district are available in Additional file 2.

**Table 3:** Incremental results of scenarios compared to baseline

	4 weeks earlier (IQR)
Cases averted	10,257 (4353–18,813)
Deaths averted	8835 (3766–16,316)
DALY s averted (thousand)	455.8 (194.1–841.11)
DALYs averted by preventing Ebola episodes (time spent with Ebola times number of cases)	0.23 (0.1–0.41)
DALYs averted by preventing premature deaths (deaths averted times remaining health adjusted life expectancy)	455.57 (194–840.7)
Costs saved (million US$)	202.82 (87.42–373.86)
Within health care sector: Ebola treatment	1.77 (0.86–2.52)
Outside healthcare sector: productivity losses	201.05 (86.56–371.34)

Median and interquartile range based on outcomes produced with the stochastic model

**Fig. 3:**
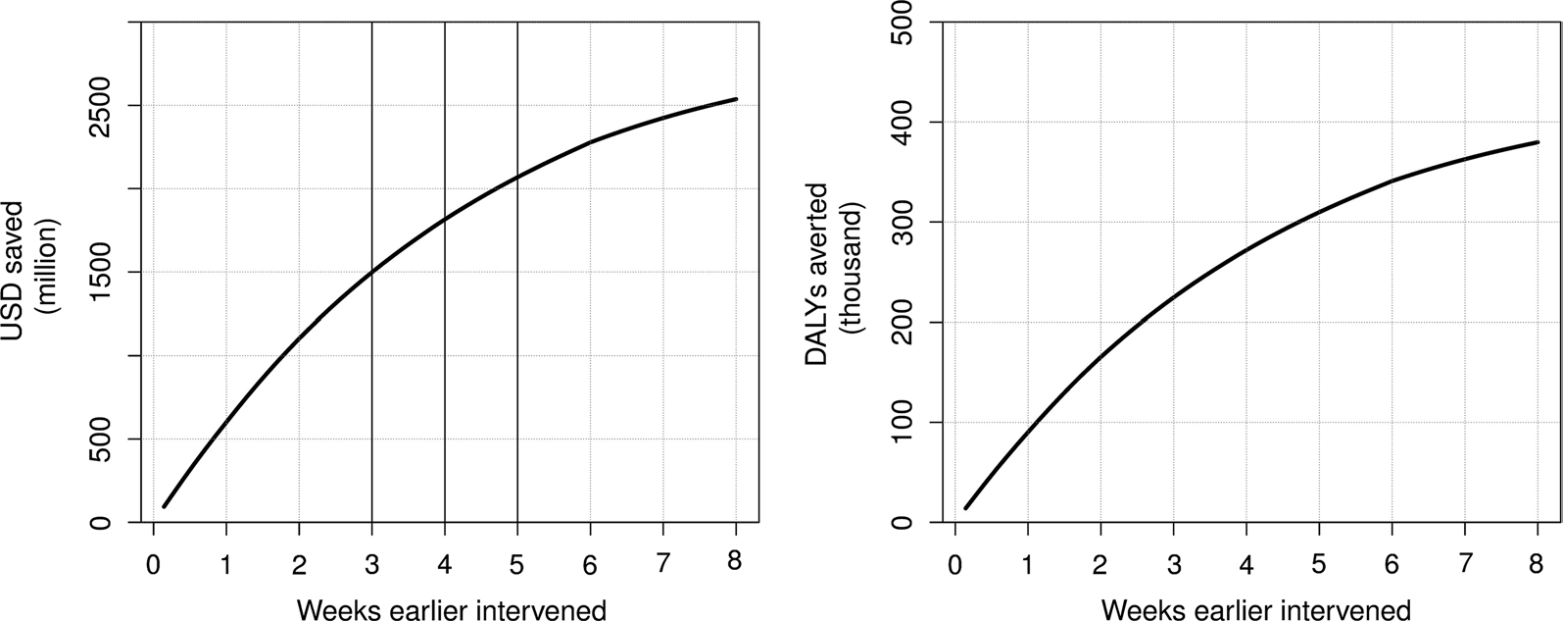
Benefits of earlier interventions in one-day increments based on outcomes produced with the deterministic model. Left-hand panel shows the costs saved, right-hand panel shows the DALYs gained

Figure 3 shows the incremental benefits of intervening earlier, from 1 day to 8 weeks, using the deterministic model. At 4 weeks, the same number of days earlier as in our main scenario, the estimated benefits gained from earlier interventions were estimated to 182 million US$. One week later would have averted 32 million US$ and 47 thousand DALYs less. Conversely, implementation 1 week earlier would yield an additional 25 million US$ and 38 thousand DALYs gained. Beyond our main scenario intervention date, the incremental benefits are diminishing in returns. Note that the average outcomes of the stochastic model as displayed in Table 3 differ from the outcomes produced with the deterministic model given the non-linearities in the model. Therefore, the numbers in Fig. 3 differ somewhat of those reported in Table 3.

**Fig. 4:**
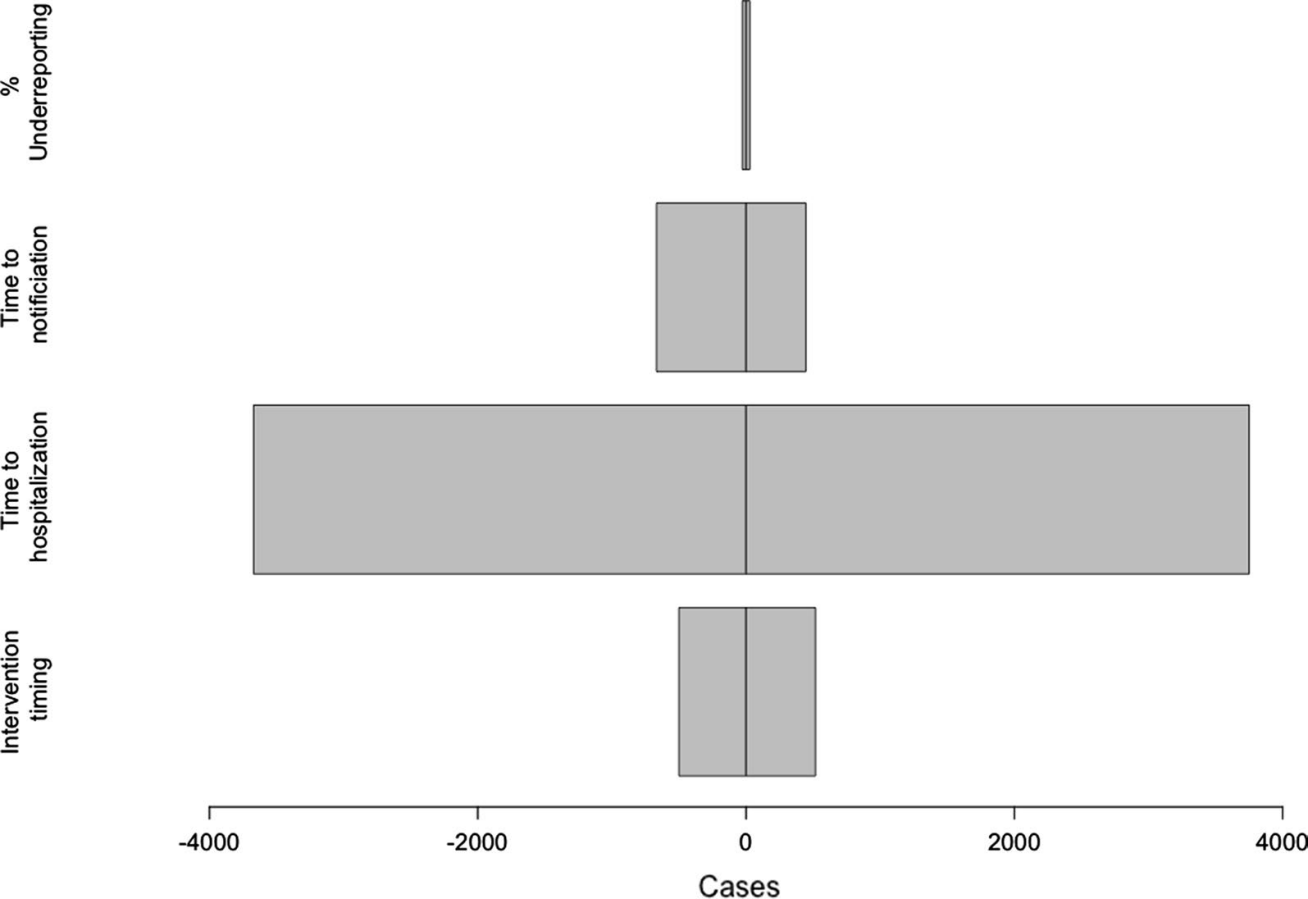
Sensitivity analysis of key parameters based on outcomes produced with the deterministic model. The parameters of interest are located on the y-axis and difference in cases compared to the baseline scenario on the x-axis. Estimates are on the left-hand side varied with ten percentage points less for the percentage of underreported, 1 day less for the time to notification, time to hospitalization and time of intervention. Right-hand side shows the difference in cases from an increase of the same amounts for the same parameter values

From the univariate sensitivity analysis, presented in Fig. 4, we found that the parameter with the greatest impact is time to hospitalization. Reducing the time of intervention by 1 day would avoid 500 cases and reducing the time to hospitalization by 1 day would avoid 3671 cases, for the time to notification the estimate is 668 cases avoided. When decreasing the underreporting by one percentage point it showed a smaller effect of 28 cases avoided. The relative decrease in values is substantially larger for the time to notification and hospitalization than for the timing of interventions.

## Discussion

This paper estimated the costs and health losses of the EVD outbreak in Sierra Leone from a societal perspective and provided estimates of the benefits from earlier interventions. The results suggest that timely interventions can reduce the loss of health and drastically reduce the economic impact of outbreaks. This emphasizes the importance of timely interventions. The largest contribution to the total cost in all scenarios was productivity losses, which arise from mortality at a young age. In our deterministic analysis, we showed that much benefit may be gained by even earlier interventions, albeit at a diminishing rate.

Before we highlight some implications of our findings, we note some limitations of this study. Importantly, several assumptions had to be made due to lacking data or poor quality data. Models previously used for EVD (e.g. [43]), allowed for explicit modeling of several transmission routes. To avoid fitting several transmission parameters and identifiability problems we did not model funeral transmissions or hospital transmissions explicitly. Evidently, funeral transmissions were an important driver of the outbreak and a facilitator of super-spreading events [44]. We assumed in our model that infectiousness remains the same throughout the symptomatic period, which may not be fully accurate and may rather be increasing closer to death [45]. The implication of this assumption is that we may have underestimated the benefits of earlier interventions, as the infected are hospitalized sooner after interventions and transmission rates are lower in hospitalized settings. Our model assumed homogenous mixing within compartments, meaning that all individuals have the same probability of contact. In reality, this assumption may not hold as individuals mix within their respective contact network primarily which may limit spread. For the current purpose, we did not include transmission caused by district interaction of individuals in different districts. This may again have underestimated the impact of the health gained and costs saved due to earlier interventions, as earlier interventions may prevent infected individuals from spreading the virus to other districts. Underreporting is assumed to occur during an EVD outbreak, however, few studies have provided concrete evidence of the proportion of underreporting. We, therefore, assumed a moderate estimate (compared to estimates by the CDC) whereby for each reported case, 2.5 cases were not reported [46]. As uncertainty exists regarding the interventions performed, assumptions had to be made to calculate the effects of the interventions. We assumed that the decline in transmission after the 1st of October 2014 was solely caused by the interventions, and not taking into account independent behavior which was not due to for example information campaigns or community leader engagement. We did not differentiate between different types of interventions as this was not our aim, we were interested in the total effect. However, in our sensitivity analysis we saw that time to hospitalization proved very important in limiting the number of new cases. Another limitation is in the use of a single date to account for the interventions performed by the UNMEER. This assumes that the interventions and the effects were more homogenous than in reality. Our estimate of the production losses is much larger than that of the cost of illness study by Bartsch et al. [31]. Our approach estimated the years of productivity lost due to EVD mortality as the HALE lost multiplied by average annual GDP of Sierra Leone and also included the latest data on reported cases. The total estimated economic loss in the baseline scenario mounted to 635 million US$. This is a smaller estimate than previously estimated by the World Bank (WB). The difference is due to the choice of approach, as the WB applied a macroeconomic level to determine the GDP loss in short and medium term. Our focus remained on individual costs to the health care system and the long-term production losses arising from deaths. An underexplored issue here is which approach is most suitable to estimate these productivity costs. In economic evaluations sometimes the human capital approach is replaced with the friction cost method, under the assumption that replacement of ill or deceased workers (through a reshuffling of labor or employing previously unemployed) will help to reduce total productivity costs (e.g. Brouwer et al. [47]). In countries and circumstances like the outbreak studied, it is unclear whether similar mechanisms exist and would lower productivity cost estimates. If we would assume this to be the case and production levels would be restored after 1 or 5 years, production costs would be estimated to be 7.07 (3.08–13.08) and 34.14 (14.61–63.29) million US$ respectively.

## Conclusions

The consequences of this outbreak proved devastating. However, it has been shown that EVD can be stopped in an early phase. Illustrated by the example of Nigeria, where quick response and actions managed to halt the outbreak containing the number of cases to 19 with seven deaths [48], however, this occurred at a later phase when the outbreak was known and the responders ready. Swift detection and isolation saved not only lives but was done at a cost of approximately 13 million US$ using the existing Polio surveillance infrastructure. This cost estimate is approximately 6 percent of the cost *savings* with interventions 4 weeks earlier in Sierra Leone. This study does not provide guidance on which preventive measures are best suited to preventing or limiting outbreaks. However, we do know that the virus was first discovered after several months of circulating in the population which advocates for systems capable of detecting emerging viruses before they spread more widely. The most important result from this study is that is considerable gains to be made from timely interventions, and that the losses primarily occurred outside the healthcare sector. To improve the capabilities for handling the next outbreak preferably before a new outbreak occurs. Timeliness is not only important in intervening, but also in the context of clear policy action.

## Supplementary information

**Additional file 1: Table S1.** District specific parameters.

**Additional file 2: Table S2.** Incremental results by district.

## Data Availability

The data supporting the findings in this study is publicly available at: https://www.who.int/csr/disease/ebola/situation-reports/archive/en/.
