# Supplementary material for: Costs and benefits of early response in the Ebola virus disease outbreak in Sierra Leone
Source: Cost Eff Resour Alloc. 2020 Mar 16;18:13. doi: 10.1186/s12962-020-00207-x (PMC7074988; doi:10.1186/s12962-020-00207-x)
Supplement: Supplementary file 1 — Additional file 1: Table S1. District specific parameters. [file 12962_2020_207_MOESM1_ESM.docx]

**Additional file 1**

Equation set 1 describes the equations governing the transmission model. In the susceptible compartment *β* is the force of infection, *φ* is the effectiveness parameter of the interventions whose value before the time of intervention is fixed to 1 and thereafter decreases. In the compartment of the latent stage (E compartment) *σ* is the time individuals spent in the phase of being infectious but not showing symptoms or being infectious to others. The proportion of *ρ* is set to move to the infectious compartment and eventually become reported cases, while the remaining proportion transitions to the infectious compartment and will not become reported cases. The I_C_ compartment represents individuals that are infectious to others but not reported. The infected compartment has a recovery rate of *γ_CR_* and the proportion *1-δ_C, while_* the proportion *δ_C_* dies at rate *γ_CD_*. The I_R0_ compartment contains those infected that will become but are not yet reported. They become reported cases at rate *ω* and die and recover at the same rate and proportion as those in the I_C_. After the transition to the I_R1,_ the infected in the model are considered reported; they die and recover at the previously mentioned proportion and rates minus the time spent in the I_R0,_ but they may be hospitalized if beds are available at rate *η*. When hospitalized, compartment H, a proportion of *1-δ_H_* individuals recover and are discharged at rate *γ_HR_*; the other proportion dies at rate *γ_HD_*. Values used from the literature are available in table 1 and estimated values are available in table S1.

| $\frac{dS}{dt}=- \frac{1}{N}(\beta\varphi I_{C}S+ \beta\varphi I_{R}S)$,  $\frac{dE}{dt}=\frac{1}{N}(\beta\varphi I_{C}S+ \beta\varphi I_{R}S)-\sigma E$,  $\frac{dI_{C}}{dt}=(1-\rho)\sigma E- (1-\delta_{C})\gamma_{CR}I_{C}-\delta_{C} \gamma_{CD}I_{C}$,  $\frac{dI_{R0}}{dt}=\rho\sigma E- \omega I_{R0}- {(1-\delta_{C})\gamma}_{CR}I_{R0}- \delta_{C}\gamma_{CD}I_{R0}$,  $\frac{dI_{R1}}{dt}=\omega I_{R0}- {(1-\delta_{C})(\gamma}_{CR}-\omega)I_{R1}- {\delta_{C}(\gamma}_{CD}-\omega)I_{R1}- \eta I_{R1}$,  $\frac{dH}{dt}=\eta I- {(1-\delta_{H})\gamma}_{HR}H- \delta_{H}\gamma_{HD}H$,  $\frac{dR}{dt}=(1-\delta_{C})\gamma_{CR}I_{C}+ {(1-\delta_{C})\gamma}_{CR}I_{R0}+{(1-\delta_{C})(\gamma}_{CR}-\omega)I_{R1}+{(1-\delta_{H})\gamma}_{HR}H$,  $\frac{dD}{dt}=\delta_{C} \gamma_{CD}I_{C}+ \delta_{C}\gamma_{CD}I_{R0}+ {\delta_{C}(\gamma}_{CD}-\omega)I_{R1}+\delta_{H}\gamma_{HD}H$, | (1) |
| --- | --- |

and the total population (N) being:

|  | $N= S+ E+ I_{C}+ I_{R0}+ I_{R1}+ R$ | (2) |
| --- | --- | --- |

And β being:

| $\beta\left( t \right)=\frac{a_{2}}{1+e^{a_{1}(t-a_{t)}}}$ | (3) |
| --- | --- |

And *φ* being:

| $\varphi\left( t \right)=\left\{ \begin{aligned} 1, &for t<intervention start \\ (1-\frac{1}{1+e^{b_{1}(t-b_{t)}}}), &for t\geq intervention start \end{aligned} \right.$ | (4) |
| --- | --- |

To allow the infection rate to vary due to reasons other than the interventions of UNMEER, the rate was modeled through a sigmoid function. The intervention efficacy was modeled as a logistic function multiplied by the transmission parameter after the date of the intervention of the 1^st^ of October 2014. The logistic function allows for a gradual implementation in both time and efficacy.

The parameter *η*, time to hospitalization among reported cases, was modeled as a linear function of time as in Kucharski *et al*. Data was gathered from WHO situation reports [1], and for months where estimates were missing, we assumed the closest value available. The values ranged from 4.6 days in the early epidemic to 1.3 days in the late epidemic. To reduce computational load, the bed capacity restraints of hospitalization was controlled through equation 5, where *H_max_* is the maximum bed capacity at a given time for a given district. When comparing the model with the term in equation 5 to the model with bed constraints modeled through roots, the two models corresponded well.

| $\eta= \eta-\frac{\eta}{{{((\hat{H}}_{t,j}+1)-H_{t,j})}^{2}}$ | (5) |
| --- | --- |

**Parameter inference**

For fitting the model, we used data from the patient database provided by the WHO website. The data are the weekly reported cases counts on a district level which we fitted against the weekly difference of the I_R1_ compartment. We fixed the following parameters with values observed by the WHO Ebola Response Team [36]. The time of the latent phase as 10.4 days, the time from onset to death in the community: 6.8 days, onset to recovery in the community: 11.7 days, onset to notification to authorities for the reported cases: 4.8 days, hospitalization to death: 5.2, hospitalization to recovery and discharge: 11.6. Time to hospitalization was modeled as a linear function using data reported by the WHO situation reports [1], resulting in a range of 4.6-1.3 days from the beginning of the outbreak to the end of the outbreak. Reported opening dates and bed numbers from the Humanitarian Data Exchange were cleaned and checked for inconsistency by comparing it to various sources such as NGOs, Situation Reports by UNMEER and Sierra Leone's Ministry of Health. In the case of fatality rates we used observed values of 60.3 percent for hospitalized cases, 91.9 percent non-hospitalized cases [32]. The model accounts for underreporting using an estimate of 83% of the cases being reported, an empirical estimate of underreporting [49]. An estimate smaller than for example the estimates in the study by Kucharski *et al* and the estimate of the CDC [13,25]. The transmission parameter was modeled as a time-dependent logistic function in order to handle the temporal heterogeneity of districts transmission. Resulting parameter values by district are available in table 2.

| **Table S1. District specific parameters** | | | | | |
| --- | --- | --- | --- | --- | --- |
| **District** | $\boldsymbol{a}_{\boldsymbol{2}}$ | $\boldsymbol{a}_{\boldsymbol{1}}$ | $\boldsymbol{a}_{\boldsymbol{t}}$ | $\boldsymbol{b}_{\boldsymbol{1}}$ | $\boldsymbol{b}_{\boldsymbol{t}}$ |
| Bo | 0,3899 | 137,0676 | -0,0037 | 0,2387 | 241,5276 |
| Bombali | 0,5067 | 242,2816 | -0,0019 | 0,0015 | 279,8948 |
| Kailahun | 0,5000 | 50,0000 | -0,0390 | 0,0127 | 739,2832 |
| Kambia | 0,5091 | 35,2471 | -0,0024 | 1,8922 | 436,6217 |
| Kenema | 0,5468 | 60,5284 | -0,0274 | 0,3494 | 741,8758 |
| Koinadugu | 0,7352 | 20,6048 | 0,0909 | 0,0978 | 173,9795 |
| Kono | 0,6307 | 299,6713 | 0,0061 | 1,9659 | 247,7990 |
| Moyamba | 0,7034 | 445,4053 | 0,0032 | 0,0005 | 741,9642 |
| Port loko | 0,4008 | 1,0003 | 0,0037 | 0,0018 | 218,6708 |
| Pujehun | 0,2204 | 160,1710 | -0,1313 | 0,6885 | 326,8079 |
| Tonkilili | 0,5686 | 56,6040 | -0,0005 | 0,0061 | 154,0043 |
| Western area rural | 0,5056 | 500,0000 | -0,0005 | 0,0181 | 251,7345 |
| Western area urban | 0,4876 | 492,1892 | -0,0004 | 0,0261 | 239,8340 |

**Remaining HALE**

We used disability weights from the GBD for suffering from EVD of 0.133 (0.088-0.19) and for a period of post EVD weights of 0.219 (0.148-0.308). The length of the period on which the post EVD weight was applied was done in a similar manner as in the Global Burden of Disease study to 0.75 years (0.417–1.135). As was the acute phase of EVD of 15.1 (14.6 – 15.6) days for recoveries and 8.2 (7.9 – 8.4) days for the deceased. From the GBD we also used remaining HALE in age groups of five years as shown in article table 2. We assumed a normal distribution from which we sampled individual HALE estimates. The lifetime production losses were estimated by multiplying the individual HALE and the annual production losses. For the production losses, we used the annual GDP per capita from the World Bank. The distribution between the age groups among the recovered and fatalities was determined by applying the observed distribution of the WHO response group [22]. Among the distribution of recovered by age groups of <15, 15-44, and ≥45 was 12.6%, 73.1%, 14.3% respectively. For deaths by age groups 14.2%, 56.5%, 29.3% respectively.
