# Supplementary material for: Costs and benefits of early response in the Ebola virus disease outbreak in Sierra Leone
Source: Cost Eff Resour Alloc. 2020 Mar 16;18:13. doi: 10.1186/s12962-020-00207-x (PMC7074988; doi:10.1186/s12962-020-00207-x)
Supplement: Supplementary file 2 — Additional file 2: Table S2. Incremental results by district. [file 12962_2020_207_MOESM2_ESM.docx]

# Additional file 2.

| Table S2. Incremental results by district | | | | | | | | |
| --- | --- | --- | --- | --- | --- | --- | --- | --- |
| District | Cases averted | Deaths averted | DALYs averted (thousands) | DALYs averted by preventing Ebola episodes | DALYs averted by preventing premature deaths | Costs saved (USD million) | Within healthcare sector | Outside healthcare sector |
| Bo | -15 (16 - -45) | -4 (12 - -40) | -0,21 (0,59 - -2,14) | 0 (0 - 0) | -0,21 (0,59 - -2,14) | -0,13 (0,3 - -0,93) | 0 (0 - -0,01) | -0,13 (0,3 - -0,92) |
| Bombali | 1202 (827 - 1662) | 1023 (697 - 1406) | 52,75 (35,89 - 72,44) | 0,03 (0,02 - 0,04) | 52,72 (35,87 - 72,39) | 24,11 (16,26 - 33,08) | 0,32 (0,22 - 0,45) | 23,79 (16,04 - 32,63) |
| Kailahun | 32 (6 - -32) | 30 (4 - -14) | 1,67 (0,19 - -0,69) | 0 (0 - 0) | 1,67 (0,19 - -0,69) | 0,58 (0,11 - -0,42) | 0 (0,01 - 0) | 0,58 (0,1 - -0,42) |
| Kambia | 20 (1 - 42) | 12 (8 - 52) | 0,58 (0,32 - 2,59) | 0 (0 - 0) | 0,58 (0,32 - 2,59) | 0,41 (0,14 - 0,96) | 0,01 (0 - 0,01) | 0,4 (0,14 - 0,95) |
| Kenema | 1 (1 - 2) | 2 (-1 - 2) | 0,09 (-0,05 - 0,06) | 0 (0 - 0) | 0,09 (-0,05 - 0,06) | 0,02 (0 - 0,08) | 0 (0 - 0) | 0,02 (0 - 0,08) |
| Koinadugu | 48 (20 - 77) | 39 (17 - 72) | 1,94 (0,87 - 3,67) | 0 (0 - 0) | 1,94 (0,87 - 3,66) | 0,87 (0,38 - 1,66) | 0,01 (0,01 - 0,01) | 0,85 (0,37 - 1,65) |
| Kono | -26 (3 - -46) | -20 (7 - -46) | -1,03 (0,35 - -2,44) | 0 (0 - 0) | -1,03 (0,35 - -2,44) | -0,48 (0,04 - -0,94) | -0,01 (0 - -0,01) | -0,48 (0,03 - -0,93) |
| Moyamba | 321 (219 - 444) | 284 (193 - 393) | 14,57 (9,95 - 20,28) | 0,01 (0 - 0,01) | 14,57 (9,94 - 20,27) | 6,45 (4,33 - 8,88) | 0,03 (0,02 - 0,05) | 6,42 (4,31 - 8,83) |
| Port Loko | 2892 (1493 - 4665) | 2528 (1277 - 4069) | 130,55 (66,07 - 210,09) | 0,06 (0,03 - 0,1) | 130,49 (66,03 - 209,98) | 56,95 (29,01 - 91,47) | 0,44 (0,23 - 0,7) | 56,51 (28,78 - 90,77) |
| Pujehun | 3 (0 - 2) | 2 (0 - 2) | 0,11 (0 - 0,08) | 0 (0 - 0) | 0,11 (0 - 0,08) | 0,03 (0 - 0,03) | 0 (0 - 0) | 0,03 (0 - 0,03) |
| Tonkolili | 900 (723 - 1104) | 785 (636 - 960) | 40,48 (32,76 - 49,55) | 0,02 (0,02 - 0,02) | 40,46 (32,74 - 49,52) | 17,86 (14,41 - 21,89) | 0,11 (0,08 - 0,14) | 17,75 (14,33 - 21,76) |
| W. A. Rural | 1436 (654 - 3704) | 1191 (570 - 3166) | 61,6 (29,25 - 163,35) | 0,04 (0,02 - 0,09) | 61,57 (29,24 - 163,26) | 29,71 (13,94 - 71,62) | 0,39 (0,18 - 0,8) | 29,31 (13,76 - 70,83) |
| W. A. Urban | 3445 (392 - 7233) | 2962 (347 - 6294) | 152,72 (17,9 - 324,27) | 0,08 (0,01 - 0,14) | 152,65 (17,89 - 324,13) | 66,45 (8,51 - 146,46) | 0,47 (0,1 - 0,37) | 65,98 (8,41 - 146,1) |
